# COVID-19-associated mortality across the countries of the Gulf Cooperation Council and how it compares to Europe: A comparative study

**DOI:** 10.5339/qmj.2021.28

**Published:** 2021-09-24

**Authors:** Ahmad Rimawi, Asem Rimawi

**Affiliations:** ^1^University of Jordan, Faculty of Medicine, Amman, Jordan; ^2^Division of Cardiology, Department of Internal Medicine, Saint Mary's Medical Group, Evansville, IN 47714 USA

**Keywords:** COVID19, Case Fatality Rate, Europe, Middle East, Pandemics

## Abstract

Introduction: In late 2019, a novel strain of coronavirus, discovered in the city of Wuhan, China, was found to cause a disease later named coronavirus disease 2019, or COVID-19. In January 2020, COVID-19 first reached the Gulf region. Afterwards, the disease spread rapidly across the countries of the Gulf and the number of COVID-19 cases rose significantly. Now, more than a year later, there are only a limited number of studies regarding COVID-19 and its behavior in this region. In this article, we aim to assess the mortality caused by the disease in the Gulf region by calculating the Case Fatality Rates (CFR) for all of the Gulf Cooperation countries and comparing the results with those of Europe.

Methods: Data was obtained from the official statistics of the World Health Organization (WHO) from January to May 2020. From the data, the CFR was calculated for every Gulf and European country included in the study. Following the calculation, the results were compared and analyzed. To make our comparison more accurate, we added the total number of COVID-19 tests per 1000 population and the Health Access and Quality index for each individual country.

Results: CFRs in the Gulf region *to May 12, 2020* were: United Arab Emirates (1.06%), Kuwait (0.69%), Saudi Arabia (0.62%), Oman (0.45%), Bahrain (0.15%), and Qatar (0.06%). Within Europe over the same time period, 10 countries had CFRs above 10%, with the majority above 3%.

Conclusions: Compared to Europe, the COVID-19 mortality rate in the Gulf region has been much lower. The difference in age groups between the Gulf region and Europe may be the most important factor, mainly due to a younger population and a smaller elderly demographic in the Gulf region. Although age is a strong factor for the lower CFR in the Gulf, other factors must also be considered. These include the number of COVID-19 tests conducted per population, different country capabilities, and varying criteria for reporting COVID-19 deaths(Table–1)(Table–2).

## Introduction:

In December 2019, a novel coronavirus910 was discovered in the city of Wuhan, China. The virus was named SARS-CoV-2 and the disease it caused COVID-19.^1^ On March 11, 2020, the WHO declared COVID-19 a global pandemic.^2^ By mid-June, it had spread to more than 200 countries, sickening 8.5 million people and causing nearly 450,000 deaths.^3^

Early in the pandemic, China, Italy and Iran were the most impacted by the virus.^4,5^ It spread rapidly in Iran, infecting more than 6,000 people by March and “200,000 on 19 June, while the number of deaths recorded reached over 10,000” by June 25.”^6^

Due to their geographical proximity to Iran, the countries of the Gulf were at a high risk for transmission of the disease. Countries of the Gulf Cooperation Council (GCC) include Saudi Arabia, United Arab Emirates (UAE), Kuwait, Qatar, Bahrain, and Oman. The virus first arrived in the Gulf region on the 29^th^ of January and the first GCC country to report a case of COVID-19 was the UAE.^7^ By February, Saudi Arabia, Kuwait, Oman and Qatar had all reported cases linked to travelers returning from Iran.^8^ By this time, multiple epidemiological studies regarding COVID-19 mortality rates in Europe and China had been reported.^9^ However, despite having a large number of confirmed COVID-19 cases, only a limited number of studies have been reported from the GCC countries regarding the mortality rates and behavior of the virus in this region.

The Case Fatality Rate (CFR) of COVID-19 is an important indicator of disease severity. It measures the proportion of cases within the total infected population who die from the disease.^10^ Given its importance, the authors aim to calculate the CFR of COVID-19 across the GCC countries. In order to strengthen the results of the study, the CFR of the European countries will be calculated for comparison. The comparison will assess the different trends in mortality across the two regions and draw accurate conclusions regarding the mortality associated with COVID-19 in the Gulf.

## Methods:

Official Statistics of the COVID-19 infection in the countries of the GCC and Europe were extracted from the WHO COVID-19 Weekly Epidemiological Update and Weekly Operational Update.^11^ The data included in this analysis were the total numbers of confirmed cases and deaths for every individual country until the 12^th^ of May 2020. Only European countries with more than 1000 total confirmed cases were included in this analysis. The data extracted was then assembled in Table 1 for the Gulf Cooperation Countries and Table–2 for the European countries. A simple analysis was then performed on the data to calculate the CFR for each country. The following formula was used to calculate the CFR:

CFR (%) =  (number of deaths due to COVID-19/total number of confirmed cases of COVID-19)  ×  100.

To make our analysis and comparison as accurate and valid as possible, we added the cumulative number of COVID-19 tests conducted per 1000 population for every individual country until May 17^th^.^12^ We also included the Healthcare Access and Quality Index (HAQ) for every individual country. The HAQ is a measure of how well developed the healthcare system is in an individual country. It is measured on a scale from 0 (worst) to 100 (best).^13^ Both the cumulative number of COVID-19 tests and the HAQ for every country were added (Table 1) and (Table 2). After calculating the CFR, we observed how they differed between the GCC and Europe and attempted to identify the main factors accounting for any differences in the CFR across the two regions.

## Results:

Among the Gulf Cooperation countries, the total number of confirmed COVID-19 cases was highest in Saudi Arabia and lowest in Oman. All the Gulf countries had a significantly low CFR of less than or equal to 1%. The UAE had the highest CFR among the Gulf countries (1.06%), while Bahrain and Qatar had an encouragingly low CFR of 0.15% and 0.06%, respectively.

Ten European countries had CFR above 10%, with the highest CFR calculated in France (19.35%). Most of the other European countries had variable CFR, though, for the majority, the CFR was above 3%. Iceland and Belarus were the only European countries with CFRs similar to that of the Gulf Cooperation countries.

## Discussion:

We can observe from the data presented that the mortality associated with COVID-19 is relatively low in the Gulf region. Moreover, comparing individual GCC and European countries with similar numbers of confirmed COVID-19 cases showed that the mortality was 5–10 times higher in Europe. Even outside Europe, only a few countries reported such low CFR.^14^ Nearby Iran, which was the source of COVID-19 transmission to the Gulf, had a CFR of 6.6% which was nearly 10 times higher than most of the GCC countries.^15^ To decrease bias in this analysis, we presented the CFR of all the European countries rather than only choosing specific countries for comparison. From the 33 European countries included in this analysis, only 2 showed CFR similar to the Gulf countries. There are many factors to consider to accurately assess the different mortality trends across the two regions. The wealth of individual countries is one of those factors since it determines how well a country can prepare and respond to COVID-19.^16^ With more resources, wealthier countries can more easily increase their capacity of critical care beds and build more field hospitals to overcome the increased demand on the healthcare system. The GCC countries in general are wealthy, with large reserves of oil and natural gas and relatively stable social systems that allow them to prepare well for such emergencies.^17^ However, the response of the United States to COVID-19 in 2020 demonstrates that the impact of wealth may be secondary to enlightened leadership and cooperative citizenry.

The onset of exposure to COVID-19 is also an important factor to assess.^18^ At the beginning of the pandemic, data regarding mortality rates and speed of transmission of the virus were scarce. Countries that were exposed earlier were less prepared and as a result were more likely to have higher CFR than others who were exposed later. Europe was exposed to the COVID-19 earlier than the GCC countries and had faster transmission rates, which might be a factor for the higher CFR across Europe.^19,20^

The level of healthcare across the GCC and Europe is also an important factor to consider in our analysis. We used the HAQ Index as an indicator for how advanced the healthcare system is in individual countries. Most of the countries in Western Europe did show advanced healthcare systems with an HAQ between 80 and 90. However, CFR of many of those countries were high with many having CFR above 10%. Across the GCC, the HAQ in general was lower than in Western Europe with only Qatar and Kuwait having similar healthcare systems. Although the healthcare systems across the GCC were less advanced than Western Europe, the CFR was much lower. We believe that although advanced healthcare systems are important for facing the current pandemic, they were not the main factor for the lower CFR across the GCC.

Testing strategies and the number of COVID-19 tests per 1000 people in individual countries was very important in our analysis. The importance of testing and its effect on the CFR stems from the need to identify all or most of the active COVID-19 cases in a specific country. As it is not possible to identify every single case in a country, countries that conduct more tests will definitely identify more COVID-19 cases than those with lower numbers of tests. More testing will also identify more asymptomatic or mild COVID-19 cases who have less severe disease and thus lower mortality rates. Therefore, countries conducting more tests will have higher denominators in the CFR equation and thus, lower CFR.^21^ This is important in the case of the GCC countries as most have conducted a large number of tests per population, allowing for more identification of active COVID-19 cases and smaller CFR in the region.

The authors attribute the low CFR in the Gulf region mainly to country demographics, which differ significantly between Europe and the Gulf. Some 19.2% of European Union citizens are older than 65 years of age.^22^ By contrast, the GCC countries have much younger populations. According to the Statistical Centre for the Cooperation Council for the Arab Countries of the Gulf, in 2016, it was estimated that only 2.2% of the residents of the GCC are above the age of 65.^23^ This large gap in age between the GCC and Europe may be the most important factor in the difference of CFR. According to data reported from China and the United States, 80% of the deaths associated with COVID-19 were among adults above the age of 60.^24^ Furthermore, the age structure of confirmed COVID-19 cases in a country has been shown to be significantly different between countries with low and high CFR.^25^ The higher mortality and increased susceptibility to severe clinical manifestations across the elderly population was observed across many studies and data sets. There are many explanations to the higher mortality in elderly patients. The higher prevalence of chronic diseases and comorbidities in the elderly population makes them more susceptible to death from the COVID-19 as a result of a decreased functional reserve that decreases their resistance to infections. Furthermore, the physiological decrease in immunity associated with aging further lowers the ability of the elderly to resist and survive infections ^26^.

There are many biases and confounding factors that may affect the results of our study. However, identifying and discussing all of them is beyond the scope of this article. Understanding the progression of the COVID-19 pandemic across different continents can help scientists and experts draw important conclusions regarding its future. The presence of evidence-based data can help policy makers adjust their current regulations regarding physical distancing, international travel and certain mitigation policies. This can have positive impacts on the economy and may accelerate the return to normal life.

### Study limitations

A major limitation to our study is that it assumes that all fatalities reported in the WHO reports are based on one known definition for a COVID-19 death. Due to the absence of clear criteria to define a COVID-19 death, different countries report deaths through different approaches. A country such as Italy may report a COVID-19 death as any person who died with a positive test regardless of whether the death was directly attributed to the COVID-19. Other countries may only report a COVID-19 death as a death that was directly caused by the virus, such as a person who developed pneumonia or acute respiratory distress syndrome. Countries with stricter definitions for COVID-19 death will report fewer deaths and consequently lower CFR.^21^ By including a large number of countries in our analysis, we believe that the differences in reporting across countries actually prove to have a minimal effect on the lower CFR across the various GCC countries. A second limitation to our study is the approach we used to estimate the CFR. While our approach provides a snapshot of the CFR in the GCC according to the data reported until the 12^th^ of May 2020, it ignores the presence of the incubation period of the virus. A proportion of the confirmed COVID-19 cases reported within this week may have not yet undergone the active disease process of the infection. The mortality of these cases will only appear after the incubation period has finished, leaving the outcome of their disease out of our study. An alternative to this approach was to insert a 14-day lag period when calculating the CFR. This approach uses the total number of confirmed COVID-19 cases at a specific date in the denominator and in the nominator, the total number of COVID-19 deaths reported after 14 days of that specific date. While this approach considers the incubation period of the virus, it is associated with many limitations. The main limitation associated with this method is that it may overestimate the true mortality caused by COVID-19 alone as patients may die from other reasons during this period.^27^ Unfortunately, across the literature, there was no consensus on the best way to estimate the CFR and we decided to use our approach due to its simplicity.^28^

## Conclusion

The mortality associated with COVID-19 was very low in the GCC countries. All of the GCC countries showed a CFR of less than or equal 1%. In countries such as Bahrain and Qatar, the CFR was encouragingly low. Despite having a high number of COVID-19 cases, *the observed mortality across the GCC was relatively low.* We believe that a young population and a high number of tests per population are the most important factors for the low mortality in the GCC countries.

### Abbreviations

**CFR:** Case Fatality Rate

**GCC:** Gulf Cooperation Council

**UAE:** United Arab Emirates

**HAQ:** Health Access and Quality Index

### Conflict of interest

The authors declare no conflict of interest.

### Author contributions

**Ahmad Rimawi:** Ideas, data collection, data analysis, manuscript writing, and editing.

**Asem Rimawi:** Critical revision of intellectual content

## Figures and Tables

**Table 1:** Extracted from WHO Weekly Epidemiological Update and Weekly Operational Update on 12/5/2021.

**Country**	**Total Cases**	**Total Deaths**	**Case Fatality Rate**	**Total Number of COVID-19 Tests per 1,000 People**	Healthcare Access and Quality Index

Saudi Arabia	41,014	255	0.62%	19.89	79.4

United Arab Emirates	18,878	201	1.06%	186.34	72.2

Kuwait	9,286	65	0.69%	60.02	82

Qatar	23,623	14	0.06%	59.16	85.2

Bahrain	5,236	8	0.15%	150.23	79

Oman	3,721	17	0.45%	N/A	77.1


COVID-19 related data for individual Gulf countries. N = 6

**Table 2:** Extracted from WHO Weekly Epidemiological Update and Weekly Operational Update on 12/5/2021.

**Country**	**Total Cases**	**Total Deaths**	**Case Fatality Rate**	**Total Number of COVID-19 Tests per 1,000 People**	**Healthcare Access and Quality Index**

Spain	227,436	26,744	11.75%	47.51	89.6

Italy	219,814	30,739	13.98%	52.46	88.7

Germany	170,508	7,533	4.42%	43.35	86.4

United Kingdom	223,064	32,065	14.37%	35.55	84.6

France	137,491	26,600	19.35%	N/A	87.9

Belgium	53,449	8,707	16.29%	66.78	87.9

Netherlands	42,788	5,456	12.75%	17.53	89.5

Switzerland	26,670	3,256	12.2%	41.94	91.8

Portugal	27,679	1,144	4.13%	69.56	84.5

Ireland	23,135	1,467	6.34%	60.89	88.4

Austria	15,874	620	3.91%	42.15	88.2

Sweden	26,670	3,256	12.20%	N/A	90.5

Poland	16,326	811	4.97%	16.33	79.6

Romania	15,588	972	6.24%	17.28	74.4

Denmark	10,513	533	5.07%	86.84	85.7

Norway	8,106	224	2.76%	41.95	90.5

Czech Republic	8,176	282	3.45%	35.51	84.8

Serbia	10,176	218	2.14%	28.93	75.4

Belarus	23,906	135	0.56%	41.03	74.4

Ukraine	16,023	425	2.65%	5.68	72.7

Finland	5,984	271	4.53%	30.36	89.6

Luxembourg	3,888	101	2.60%	102.65	89.3

Republic of Moldova	4,995	179	3.58%	N/A	73.1

Greece	2,726	151	5.54%	13.38	87

Hungary	3,313	425	12.83%	14.77	79.6

Croatia	2,196	91	4.14%	13.89	81.6

Iceland	1,801	10	0.56%	170.5	93.6

Estonia	1,741	61	3.50%	55.57	81.4

Lithuania	1,485	50	3.36%	90.21	76.6

Slovenia	1,460	102	6.99%	35.05	87.4

Bosnia and Herzegovina	2,142	112	5.23%	N/A	78.2

North Macedonia	1,664	91	5.47%	10.93	76

Slovakia	1,457	26	1.78%	27.8	78.6


COVID-19 related data for individual European countries. N = 33
